# Supplementary material for: Mortality, Morbidity and Health-Related Outcomes in Informal Caregivers Compared to Non-Caregivers: A Systematic Review
Source: Int J Environ Res Public Health. 2022 May 11;19(10):5864. doi: 10.3390/ijerph19105864 (PMC9141545; doi:10.3390/ijerph19105864)
Supplement: Supplementary file 1 [file ijerph-19-05864-s001.zip › ijerph-1662324-supplementary.pdf]

Supplement: Search Strategy

Table S1

Database	Search terms	Results	Filters
1. PubMed	#1: ("Home Nursing"[Mesh])	1,259	Filters: last 10 years
2. CINAHL	#1: (MH "Home Nursing")	1,448	Limiters - Published Date: 20090101-20201231
3. Web of Science	#1: TS=<br>(”informal caregiver*”<br>OR “home nursing” OR<br>“nursing relatives” OR<br>“caring relatives” OR<br>“caregiving relatives” OR<br>“family caregivers”)<br><br>#2: TS=<br>(disease* OR<br>illness OR<br>burden OR<br>strain OR<br>health OR<br>satisfaction OR<br>“quality of life” OR<br>effect)<br><br>#3: TS= (intervention OR<br>child* OR<br>palliative)<br><br>#1 AND #2 NOT #3	 3,175<br>1,062<br>3<br>27<br>18<br>6,714<br><br><br>5,424,140<br>298,207<br>207,941<br>1,132,492<br>2,178,751<br>161,166<br>273,397<br>6,165,277<br><br>685,689<br>1,108,097<br>44,990<br><br>2,824	Filters: last 10 years

Conducted by P.J.
